# Supplementary material for: Time Course RNA-seq Reveals Soybean Responses against Root-Lesion Nematode and Resistance Players
Source: Plants (Basel). 2022 Nov 4;11(21):2983. doi: 10.3390/plants11212983 (PMC9655969; doi:10.3390/plants11212983)
Supplement: Supplementary file 1 [file plants-11-02983-s001.zip › Supplementary Figures S1-S5.pdf]

Time Course RNA-seq Reveals Soybean Responses Against Root-Lesion Nematode and Resistance Players

Valéria Stefania Lopes-Caitar [1,2], Rafael Bruno Guayato Nomura [2,3], Suellen Mika Hishinuma-Silva [2,3], Mayra Costa da Cruz Gallo de Carvalho [4], Ricardo Vilela Abdelnoor [2], Waldir Pereira Dias [2] and Francismar Corrêa Marcelino-Guimarães [2,*]

[1] Department of Biological Sciences, Universidade Estadual de Londrina (UEL), Londrina 86057-970, PR, Brazil
[2] Brazilian Agricultural Research Corporation-Embrapa Soja, Londrina 86001-970, PR, Brazil
[3] Department Biochemistry and Biotechnology, Universidade Estadual de Londrina (UEL), Londrina 86057-970, PR, Brazil
[4] Department of Biological Sciences, Universidade Estadual do Norte do Paraná (UENP), Bandeirantes 86360-000, PR, Brazil
* Correspondence: francismar.marcelino@embrapa.br

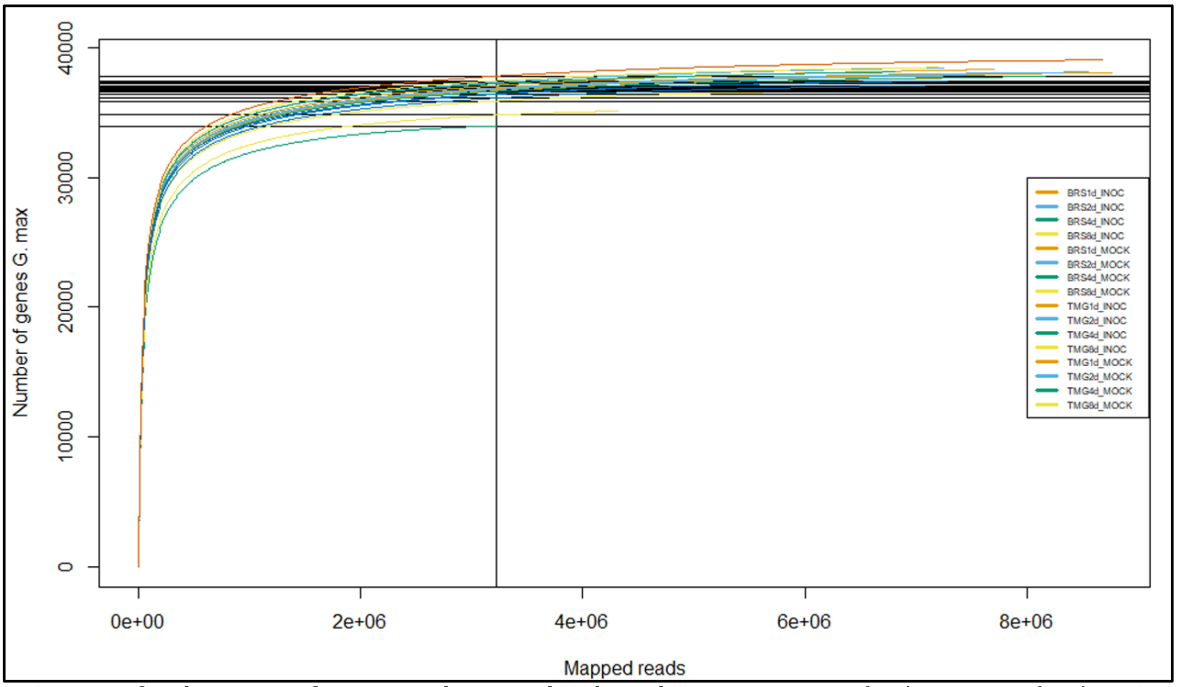

Figure S1. Rarefaction curve for transcriptome depth and coverage. Rarefaction curves for the 16 RNA-seq libraries, representing number of mapped genes in soybean genome.

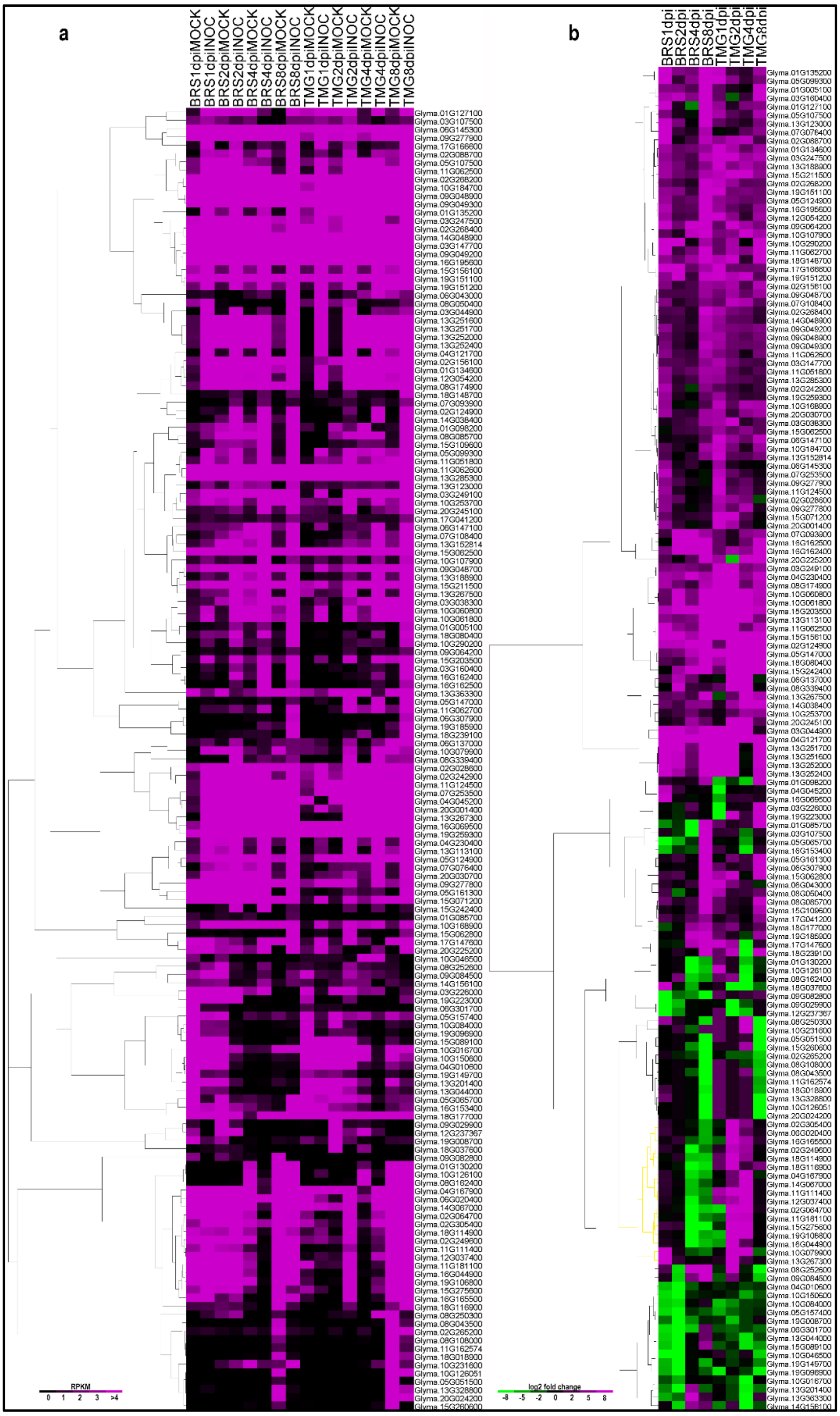

Figure S2. Heatmap of the 157 co-expressed genes among each genotype time-points and/or between genotypes. Set of co-expressed genes hierarchical clustered by RPKM values (a). Set of co-expressed genes hierarchical clustered by fold change values. From the top to the middle of the figure are branches of genes that presented similar up-regulation profile. The branch of similar opposite co-expressed between genotypes is highlighted in yellow. On the bottom of the figure are branches of genes that presented similar down-regulation profile (b).

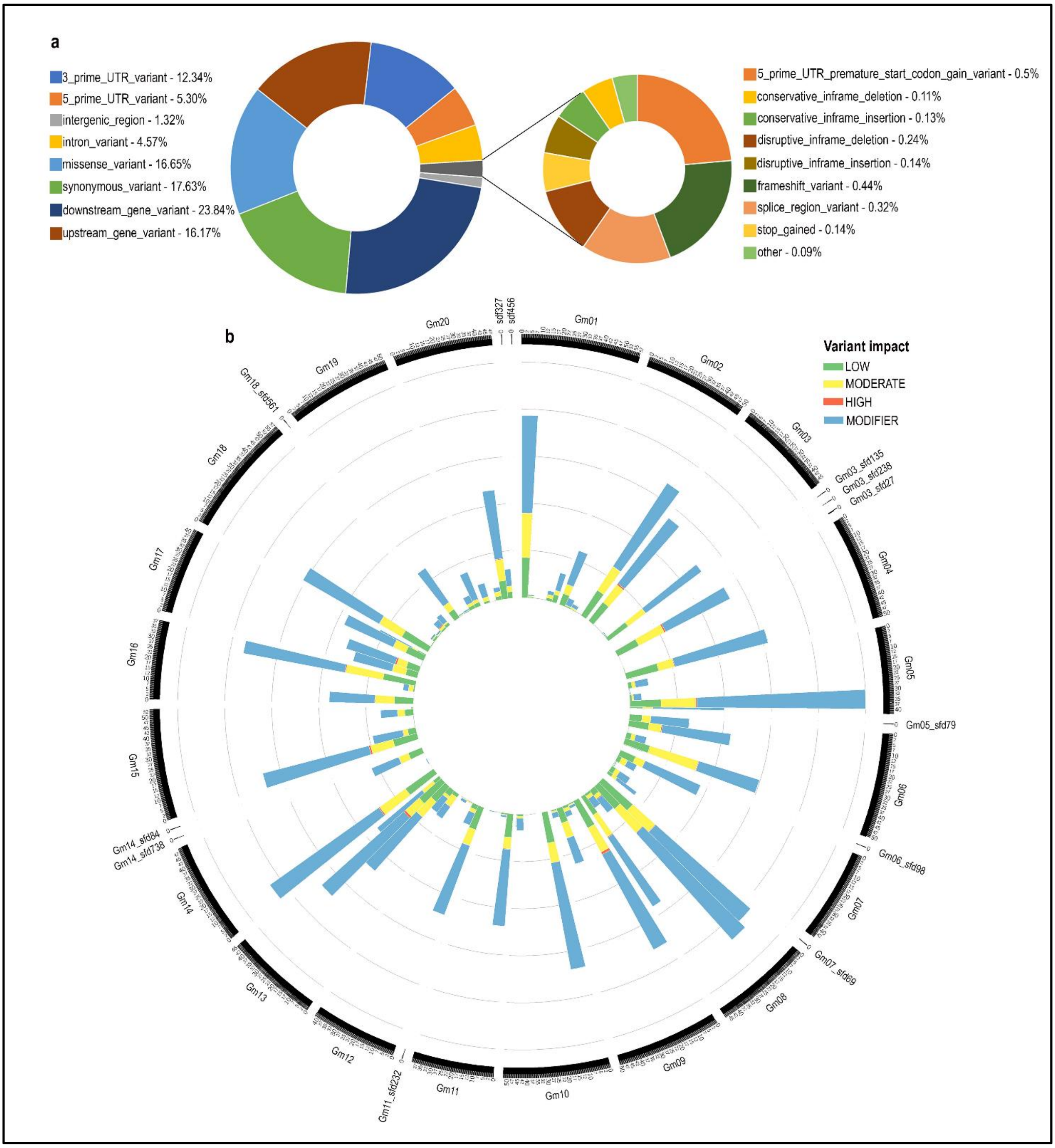

Figure S3. Exclusive BRS variants identified in transcripts region after comparison with TMG and Williams 82. Donut chart shows variants distribution in genomic BRS regions (a). Circle stacked histogram with the organization of variants on soybean genome based on impact level – 500 kbp interval (b).

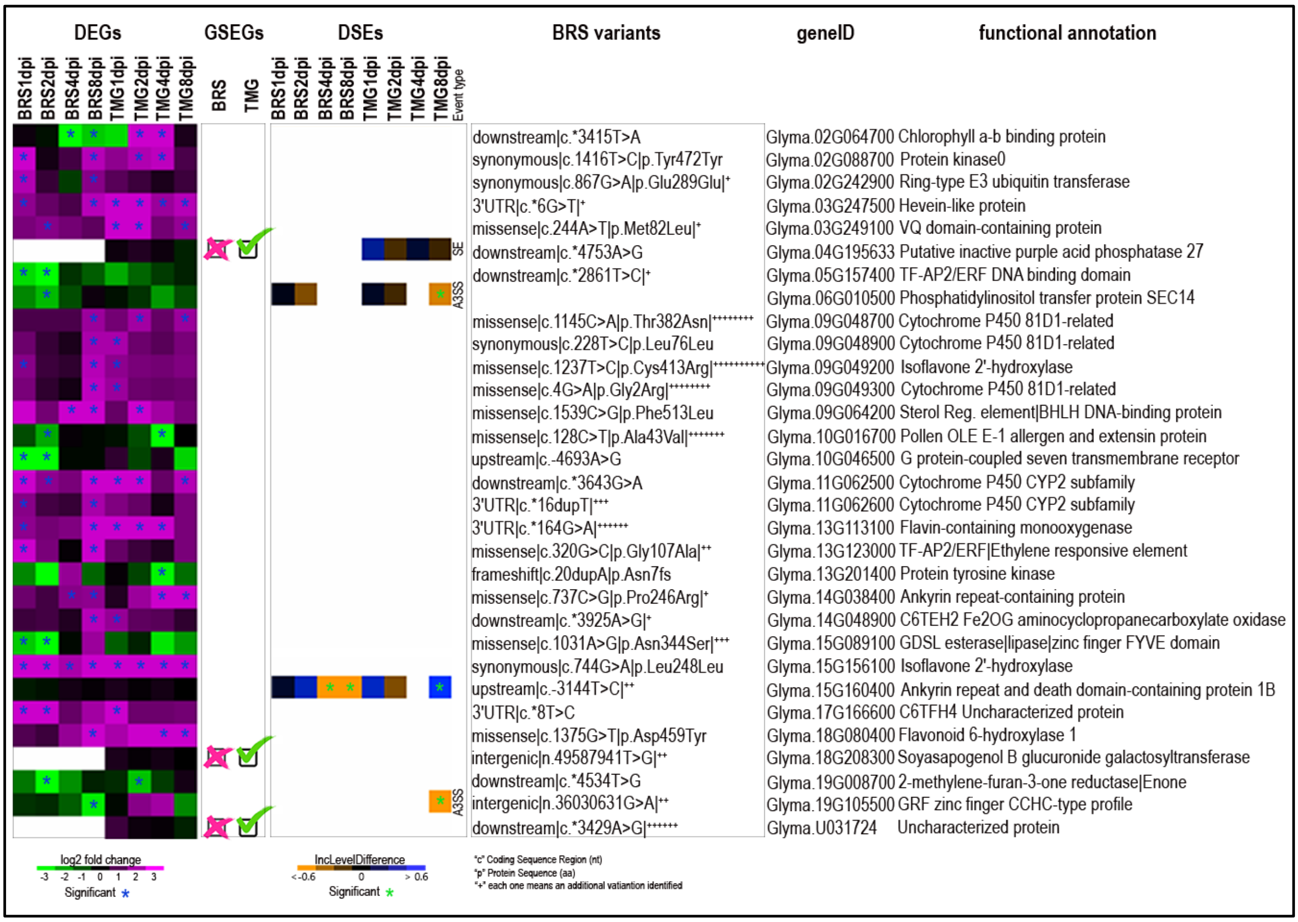

Figure S4. Combination results of genes identified in more than one analysis. Genes can present more than one genome variant in BRS.

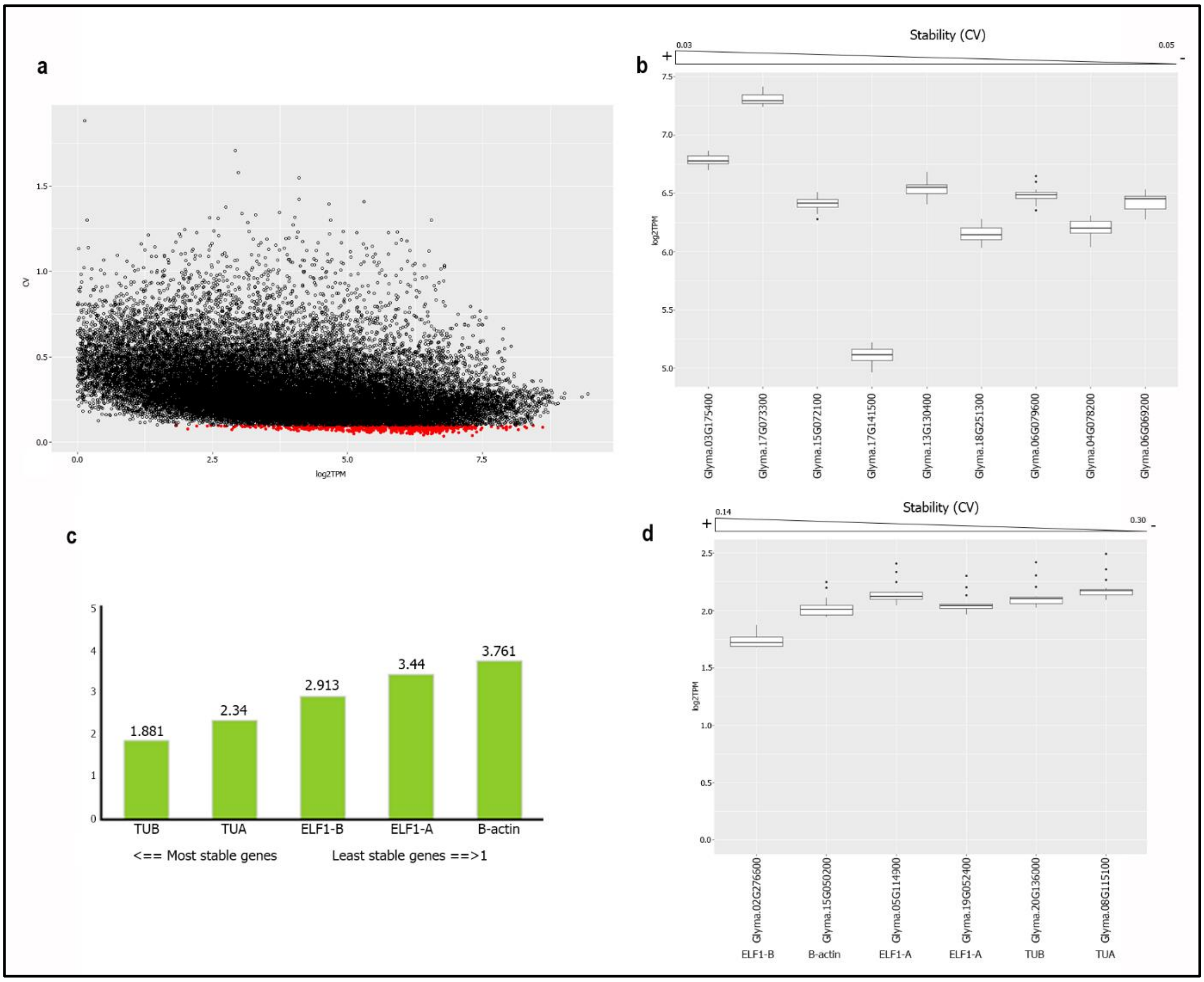

Figure S5. Analyses of soybean gene expression stability under RLN infection. Scatterplot of the coefficient of variation (CV) against average expression values (TPM) after log2 transformed based on RNA-Seq data (n = 16). Each circle represents a gene, and dark-red circles highlight genes with CV < 0.1 (a). Boxplot of the top 10 most stable genes identified on RNA-seq analyses presenting the lowest CV and average TPM > 5. Genes showing the lower CV values (b). Graphic bars of soybean commonly used reference genes and their stability profile in our RNA samples, obtained by qPCR, based on the geometric mean of classification, generated by ReFinder (c). Boxplot soybean commonly used reference genes and their stability profile based on soybean-RLN RNA-seq data (d).

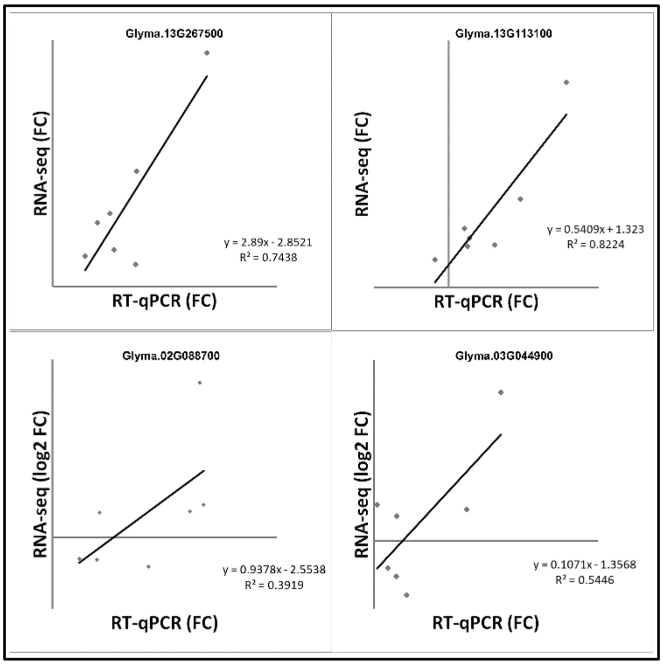

Figure S6. Validation of gene expression by time-point/treatment. Relative expression of seven selected genes displays a high correlation between RNA-seq expression data and qPCR.
